# Abnormal visual and olfactory sensations during radiation therapy: a prospective study

**DOI:** 10.1007/s00066-023-02095-5

**Published:** 2023-06-04

**Authors:** Yiling Mai, Celina Vogel, Julia Thiele, Tobias Hölscher, Thomas Hummel

**Affiliations:** 1 Smell and Taste Clinic, Department of Otorhinolaryngology, Faculty of Medicine Carl Gustav Carus, Technische Universität Dresden, Fetscherstraße 74, 01307 Dresden, Germany; 2 Department of Radiotherapy and Radiation Oncology, Faculty of Medicine and University Hospital Carl Gustav Carus, Technische Universität Dresden, Dresden, Germany; 3 OncoRay—National Center for Radiation Research in Oncology, Faculty of Medicine and University Hospital Carl Gustav Carus, Technische Universität Dresden, Helmholtz-Zentrum Dresden–Rossendorf, Dresden, Germany

**Keywords:** Phantosmia, Phosphene, Radiation therapy, Proton, Photon

## Abstract

**Purpose:**

Patients sometimes report phosphene and phantosmia during radiation therapy (RT). However, the detail features and related factors are not well understood. Our prospective study aimed to investigate the characteristics of phantosmias and phosphenes, to identify factors that influence the occurrence, intensity and hedonic (pleasantness/unpleasantness) ratings of such sensations during RT.

**Methods:**

We included a total of 106 patients (37 women), who underwent RT in regions of the brain, ear, nose, throat (ENT), and other areas of the body for a duration of 43 ± 5 days. Medical history and treatment parameters were collected in a structured medical interview. Olfactory function was measured using the Sniffin’ Stick Odor Identification Test at baseline. Phantosmia and phosphene were recorded weekly based on a self-report questionnaire.

**Results:**

There were 37% of the patients experiencing phantosmias, 51% experiencing phosphenes, and 29% simultaneously experiencing both sensations. Phosphenes were typically perceived as a flashily blue, white and/or purple light, phantosmias were typically perceived as a chemical-like, metallic or burnt smell. Younger age (F = 7.81, *p* < 0.01), radiation in the brain region (χ^2^ = 14.05, *p* = 0.02), absence of taste problems (χ^2^ = 10.28, *p* = 0.01), and proton RT (χ^2^ = 10.57, *p* = 0.01) were related to these abnormal sensations. History of chemical/dust exposure predicted lower intensity (B = −1.52, *p* = 0.02) and lower unpleasantness (B = 0.49, *p* = 0.03) of phantosmia. In contrast, disease (tumor) duration (B = 0.11, *p* < 0.01), food allergy (B = 2.77, *p* < 0.01), and epilepsy (B = −1.50, *p* = 0.02) influence phosphenes intensity. Analgesics intake predicted a higher pleasantness of the phosphenes (B = 0.47, *p* < 0.01).

**Conclusions:**

Phantosmias and phosphenes are common during RT. The treatment settings and individual arousal level influence the occurrence, intensity and hedonic of such abnormal sensations. Phantosmias and phosphenes may involve more central neural than peripheral mechanism, and they could be elicited with activation of areas that are not regarded to be part of the olfactory or visual network.

## Introduction

Patients sometimes report abnormal visual (i.e., phosphene) and olfactory (i.e., phantosmia) sensations during radiation therapy (RT) [1, 2]. While phantosmia indicates a phenomenon that an odor is perceived in the absence of an odorous stimulus [3], phosphene describes the phenomenon of visual sensations without light actually entering the eye [4]. It is estimated that approximately 5–68% of the patients report a phosphene [2, 5], and 4–44% of the patients report a phantosmia during RT [1, 5]. However, phantosmia and phosphene are rare among the general population and uncommon among the clinical population. Furthermore, although abnormal sensations generally do not interrupt the treatment [6], they sometimes irritate patients and may reduce their compliance [7]. Hence, to improve treatment compliance and provide adequate management, it is important to understand the clinical characteristics of these phenomena.

In recent years, emerging studies on this topic made significant contributions. Based on what has been reported, percepts of ozone, burnt sensations and chemical-like smells are typical for phantosmias [6], and blue, purple and yellow are common colors in phosphene during RT [8]. Although heterogeneous, the intensity of these unusual sensations ranges from mild to moderate, and usually would disappear after RT has been finished [1, 7, 8]. In addition, age, radiation site, dose and RT technology are regarded to be associated with the occurrence of these phenomena [6].

However, the clinical characteristics of these phenomena are not fully understood. First, previous studies have reported such phenomena, but most of them used a retrospective design which may underestimate the frequencies of phosphenes and phantosmias due to the nature of retrospective design [5]. And although there are emerging studies using a prospective design, the number of investigated cases remains small.

In addition, patients who co-experience phosphenes and phantosmias are rarely reported and investigated. Besides, data of the hedonic ratings of these sensations are limited, which is meaningful for improving patients’ compliance and preparing appropriate interventions. Furthermore, although few studies tried to identify factors that predict the occurrence of these phenomena, predictors of intensity and valence of these sensations remain unclear. Last but not least, there is limited knowledge regrading which patients with which medical conditions may be at risk of developing phosphene or/and phantosmia. For example, previous studies hypothesized that the higher degree of phantosmias in younger people could be explained by their better olfactory function compared to that of older patients [6]. However, this was only assumed, and the olfactory function of these patients had not been tested.

Considering existing knowledge and research gaps, our present study used a prospective design and aimed to (a) investigate the detailed characteristics of phantosmias and phosphenes during RT including their co-occurrence, (b) identify factors that influence the occurrence, intensity and valence of phantosmias and phosphene, including treatment factors, medical conditions, and demographic factors.

## Methods

### Participants

As part of the Visual and Olfactory (ViOl) study protocol, we prospectively enrolled all consenting patients who received proton or photon therapy (including stereotactic treatments) in the brain areas, ear, nose, and throat (ENT) areas, as well as those who underwent proton therapy in other regions of the body, at the Department of Radiotherapy and Radiation Oncology, Faculty of Medicine and University Hospital Carl Gustav Carus, Technische Universität Dresden, Dresden, Germany. Inclusion Criteria: (a) Age ≥ 18 years, (b) Planned proton or photon therapy with a locally ablative dose of radiation, (c) Verbal and Written Consent, (d) sufficient general condition (e.g., awake during radiation and able to communicate). Exclusion criteria: (a) insufficient German language skills, (b) palliative treatment concept, (c) participation in an intervention study whose procedures are inconsistent with the present study, (d) insufficient dose of radiation to qualify as curative care (a total of dose of < 40 Gray [Gy]). The patients are informed about this study and receive study-specific patient information and a declaration of consent. Only patients who had signed them were included. The design of this study was approved by the Ethics committee at the Faculty of Medicine and University Hospital Carl Gustav Carus (protocol number STR-ViOl-2019).

### Measurements

#### Medical parameters

Patients’ medical records were collected before the radiation therapy based on the patient’s self-report. Demographic data included age, gender. Pre-existing conditions included hypertension, epileptic seizures, diabetes, asthma, obesity, allergy, depression, medication intake, head trauma, surgery (nasal, ophthalmic and tumor), nasal polyp, allergy rhinitis, allergy rhinosinusitis, olfactory phantosmia earlier, eyesight, cataract, glaucoma and taste problem, medication intake. Risk factors exposures included alcohol, tobacco, dust/chemical exposure.

#### Disease and treatment parameters

Tumor disease, RT location (Brain region [not included olfactory bulb], ENT-related region, other body region), disease duration, technology of RT (protons/photons), radiation dose (Equivalent dose in 2 Gy fractions, EQD2) in general and for a single radiation, chemotherapy, and the occurrence of metastases were recorded.

#### Abnormal sensation

After completing each weekly course of RT, patients were asked to complete a questionnaire that assessed their perception of olfactory and visual sensations of each RT session throughout the week. The questionnaire collected information on the intensity of the sensation (rated on a scale of 0 to 10), its hedonic ratings ([−2 extreme unpleasant] to [0 neutral] to [+2extreme pleasant]), the description of the sensation, and how long it lasted (whether it changed after one single RT session).

#### Olfactory function

The Sniffin’ Stick Identification test [9] was used to assess the olfactory function ideally at the beginning of the radiation therapy (before radiation therapy up to a maximum of the 5th fraction). The test performed, based on a force choice task, by presenting the subjects with a single pen and ask them to identify and label the odor from four alternative descriptors for each pen. Total score was also calculated by summing all the correctly identified odors, ranging from 0 to 16 points [10].

### Data analysis

Data were analyzed using SPSS 27.0 software (IBM Corp., Armonk, NY, USA). First, descriptive analyses were conducted to show the percentage and characteristics of the patients having phosphenes and/or phantosmias during RT. And the percentage of these abnormal sensations in different radiation regions was reported. To compare the differences of clinical features between patients without or with phosphenes and/or phantosmias, group comparisons were examined. For categorical variables, Chi-square test was conducted and counts (percent) were reported. For continuous variables with normal distribution, one-way analysis of variance (ANOVA) was conducted, and mean ± standard deviation (SD) was reported. For continuous variables with non-normal distribution, Kruskal-Wallis H test was conducted, and median ± interquartile range was reported. If group sample size was less than 10, descriptive statistics of the variable would be described but further statistical analyses would not be conducted in order to avoid misleading outcomes due to small sample sizes. In addition, logistic regression analysis was used to identify predictors that predict the occurrences of phantosmia and/or phosphenes. Linear regression analyses were conducted to identify predictors for the intensity and hedonic ratings of phantosmias and phosphenes.

## Results

### Descriptive analyses

123 patients gave their consent to participate in the study. However, after evaluation, 106 participants (37 women, 69 men) with a mean age of 54.4 ± 13.4 years were included. There were 17 drop outs: 5 patients were wrongly included (e.g., they were treated in a palliative intention), 5 patients changed their mind and wanted to be excluded from the study, 3 patients quit due to a change in therapy regime or no RT at all, 3 patients were excluded because they failed to fill in questionnaires, and 1 patient dropped because of the radiation dose (only 20 Gy in total) was too low to qualify as curative care. Radiation period for all included patients ranged from 22 to 55 days, with the mean and SD of 43.3 ± 5.5 days. The averaged radiation dose in total was 62.66 ± 7.90 Gy, and the single dose was 1.95 ± 0.10 Gy. Other characteristics of each sensation group are detailed in Table 3.

**Fig. 1:**
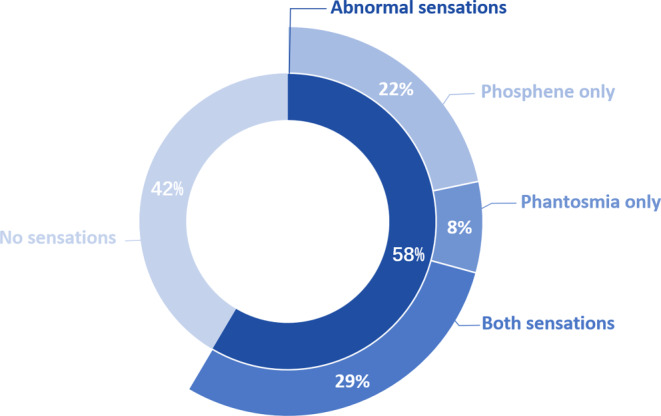
Percentage of patients experienced phantosmias and/or phosphenes. Note. The dark blue portion of the inner ring indicates the percentage of patients who experienced phantosmias and/or phosphenes during radiation therapy. The darkest, lighter, and lightest portions of the outer ring represent the percentage of patients experiencing both sensations, only phantosmia, and only phosphene, respectively

Among the entire cohort, over half of the patients (*n* = 61, 58%) experienced at least one of the abnormal sensations during RT. Phosphenes (*n* = 54, 51%) were more frequent than phantosmias (*n* = 37, 35%). In addition, more patients co-experienced two sensations (29%) than patients experienced only phantosmias (8%) or phosphenes (22%) during RT (Fig. 1).

**Fig. 2:**
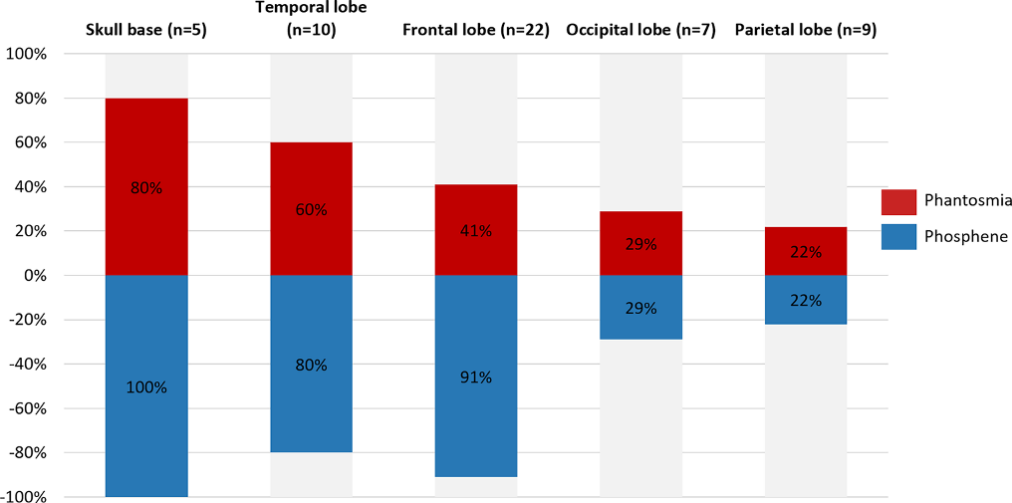
Phantosmias and phosphenes in patients who received different targeted radiation regions

The treated regions included brain and ENT-related areas, and other body regions. Targeted brain regions included frontal lobe, temporal lobe, parietal lobe, occipital lobe, cerebellum, sphenoid bone, brainstem, external/extreme capsule, pituitary and skull base. We also described number and percentage of phantosmias and phosphenes in patients who received radiation in different target regions (Fig. 2).

Results of the Sniffin’ Stick Identification test. As shown in Table 3, there were no significant differences of olfactory function (F = 0.16, *p* = 0.92) in patients with both phantosmias and phosphenes (11.83 ± 1.79), with phantosmias only (11.43 ± 1.81), with phosphenes only (11.74 ± 2.63), and without sensations (11.47 ± 2.64).

### Characteristics of phantosmias and phosphenes during the radiation therapy

**Fig. 3:**
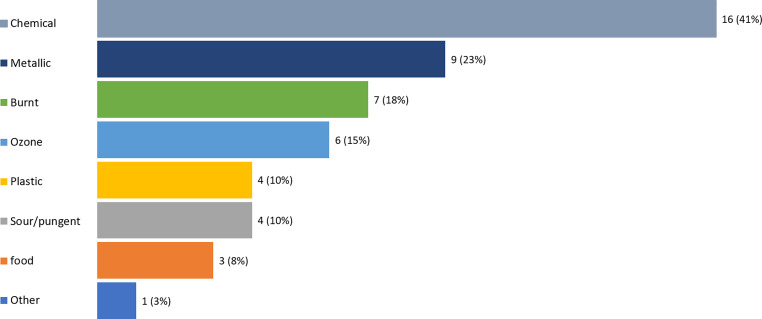
Description of phantosmias during the radiation therapy (*N* = 39)

As shown in Table 1, among 39 patients with phantosmias, 8 (21%) had phantosmias only, while the remaining 31 (79%) patients had phantosmias accompanied by phosphenes. While the mean intensity was 5.51 (SD = 1.93, t _[compared to 0]_ = 17.60, *p* < 0.01), the mean hedonic value was − 0.51 (SD = 0.67, t _[compared to 0]_ = 4.69, *p* < 0.01). The phantosmias would disappear immediately after the RT session in 87% the patients. The most typical odors were described as chemical-like (*n* = 16, 41%), metallic (*n* = 9, 23%) or burnt smell (*n* = 7, 18%). Ozone, food, plastic, sour/pungent smells were also reported occasionally (Fig. 3).

**Table 1:** Characteristics of phantosmia during the radiation therapy

	Phantosmia (*N* = 39)	Phantosmia only (*N* = 8)	Phantosmia accompanied by phosphene (*N* = 31)
Intensity (0 to 10)	5.51 ± 1.93	5.24 ± 1.36	5.58 ± 2.05
Hedonic (−2 to +2)	−0.51 ± 0.67	−0.36 ± 0.48	−0.55 ± 0.71
Change with time	34 (87.2%)	7 (87.5%)	27 (87.1%)
*Descriptions*			
Metallic	9 (23.1%)	2 (25.0%)	7 (22.6%)
Ozone	6 (15.4%)	0	6 (19.4%)
Chemical	16 (41.0%)	3 (37.5%)	13 (41.9%)
Burnt	7 (17.9%)	0	7 (22.6%)
Food	3 (7.7%)	0	3 (9.7%)
Plastic	4 (10.3%)	0	4 (12.9%)
Sour/pungent	4 (10.3%)	0	4 (12.9%)
Other	1 (2.6%)	1 (12.5%)	0

**Fig. 4:**
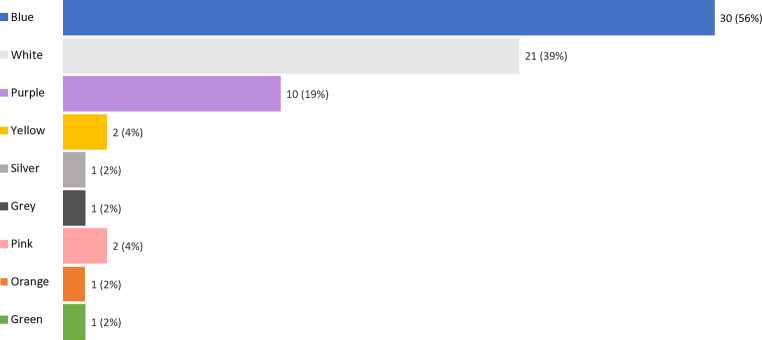
Description of phosphenes during the radiation therapy (*N* = 54)

As shown in Table 2, among 54 participants experiencing phosphenes, there were 43% (*n* = 23) who had phosphenes only, while 57% (*n* = 31) reported phosphenes accompanied by phantosmias. The mean intensity was 5.97 (SD = 1.99, t _[compared to 0]_ = 21.20, *p* < 0.01), while the mean hedonic value was −0.03 (SD = 0.39, t _[compared to 0]_ = 0.56, *p* = 0.58). Phosphenes were typically perceived as flashy light (*n* = 50, 93%) in both eyes (*n* = 37, 69%), and constant light in the left or right eye was sometimes reported. In addition, 78% (*n* = 42) of patients with phosphenes reported that the sensation changed with time. When it comes to phosphene color, blue (*n* = 30, 56%), white (*n* = 21, 39%) and purple (*n* = 10, 19%) were the three most common colors, while other colors were also reported sometimes (Fig. 4).

**Table 2:** Characteristic of phosphene during the radiation therapy

	Phosphene (*N* = 54)	Phosphene only (*N* = 23)	Phosphene accompanied by phantosmia (*N* = 31)
Intensity (0 to 10)	5.97 ± 1.99	5.32 ± 1.95	6.40 ± 1.93
Hedonic (−2 to +2)	−0.03 ± 0.39	0.17 ± 0.01	−0.06 ± 0.37
Change with time	42 (77.8%)	15 (65.2%)	27 (87.1%)
Flash of light	50 (92.6%)	19 (82.6%)	31 (100.0%)
Constant light	6 (11.1%)	5 (2.2%)	1 (3.2%)
Left eye	4 (7.4%)	4 (17.4%)	0
Right eye	6 (11.1%)	2 (8.7%)	4 (12.9%)
Both eyes	37 (68.5%)	12 (52.2%)	25 (80.6%)
*Description*			
Blue	30 (55.6%)	8 (34.8%)	22 (71.0%)
White	21 (38.9%)	9 (39.1%)	12 (38.7%)
Purple	10 (18.5%)	2 (8.7%)	8 (25.8%)
Yellow	2 (3.7%)	2 (8.7%)	0
Silver	1 (1.9%)	1 (4.3%)	0
Grey	1 (1.9%)	0	1 (3.2%)
Pink	2 (3.7%)	1 (4.3%)	1 (3.2%)
Orange	1 (1.9%)	1 (4.3%)	0
Green	1 (1.9%)	0	1 (3.2%)

#### Factors that influence the occurrence of phantosmias and phosphenes

**Fig. 5:**
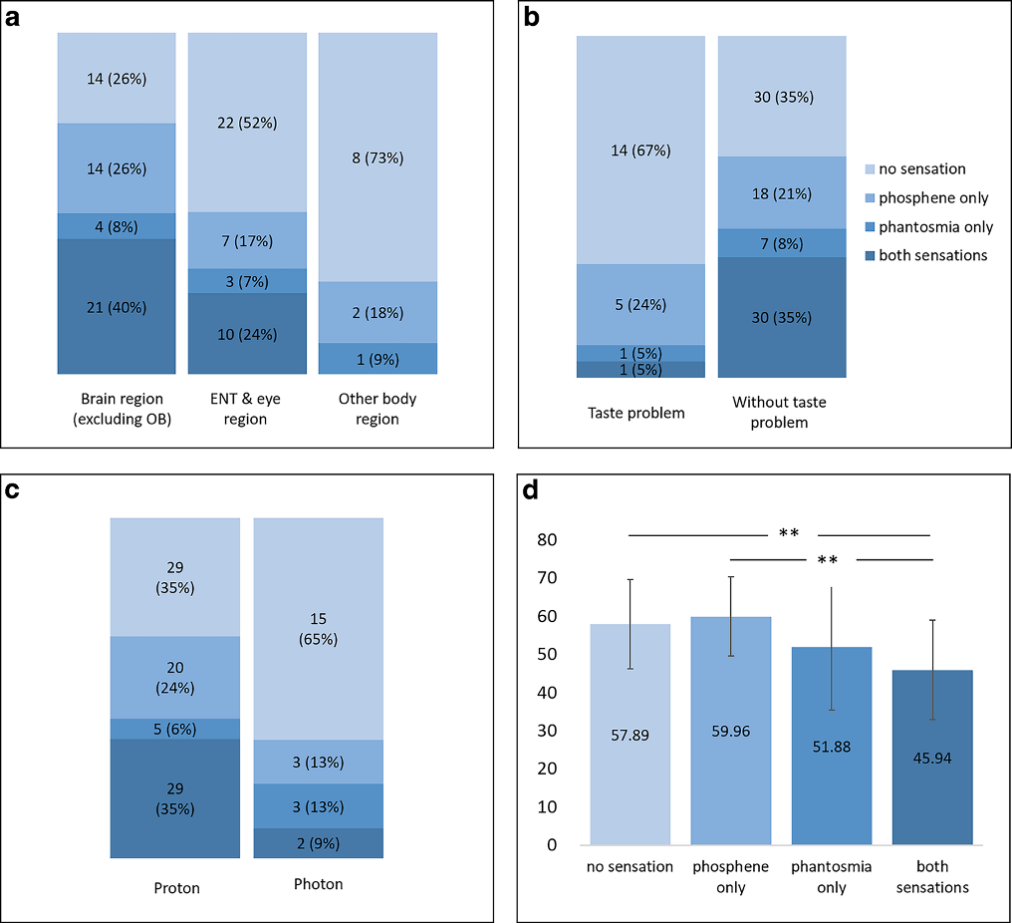
Factors that influence the occurrences of phantosmias and phosphenes. Note. **a** Targeted radiation region in different sensation groups: there were significant differences between brain vs. other body region in both sensations and no sensation group. **b** Taste problem in different sensations groups: there were significant difference between taste problem and no taste problem in both sensations and no sensation group. **c** Radiation technology in different sensations groups: there were significant differences between photon and proton in both sensations and no sensation group. **d** Age for different sensations groups

ANOVA analysis suggested that age was different between patients with abnormal sensations (F = 7.81, *p* < 0.01). Post hoc comparison suggested that patients experiencing both sensations were younger than those without sensations (95% confidence interval [CI] [4.22, 19.68], *p* < 0.01) and with phosphenes only (95% CI [4.95, 23.09], *p* < 0.01). Chi-square tests found that the radiation target region (χ^2^ = 14.05, *p* = 0.02), self-reported taste problems (χ^2^ = 10.28, *p* = 0.01), and the radiation technology (χ^2^ = 10.57, *p* = 0.01) were associated with the occurrence of phantosmias and/or phosphenes. In terms of the region of radiation, a higher percentage of patients in the general abnormal sensation group or the co-occurrence of both sensations group received RT of the brain region or ENT region than other body regions. But only RT of the brain region reached a significant level compared to body region: general abnormal sensation group, 74% vs. 27%, *p* < 0.05; co-occurrence of both sensations group: 40% vs. 0%, *p* < 0.05. Regarding taste problem, patients in the general abnormal sensation group (65% vs. 33%, *p* < 0.05) or the co-occurrence of both sensations group (35% vs. 5%, *p* < 0.05) had a significantly higher percentage of an absent taste problem than having a taste problem. As for radiation technology, a significantly higher percentage of the patients in the general abnormal sensation group (65% vs. 35%, *p* < 0.05) or the co-occurrence of both sensations group (35% vs. 9%, *p* < 0.05) were received the proton RT than the photon RT (Table 3 and Fig. 5).

**Table 3:** Descriptive analyses and group comparisons in patients experienced phantosmia and/or phosphene during radiation therapy

	No sensation (*n* = 44)	Phosphene only (*n* = 23)	Phantosmia only (*n* = 8)	Both sensations (*n* = 31)	Total (*n* = 106)	χ^2^/F	*p*
*Age*	57.9 ± 11.7	60.0 ± 10.5	51.9 ± 16.5	45.9 ± 13.1	54.4 ± 13.4	**7.81**^**a**^	**<** **0.01**
*Gender*							
Women	13 (35.1%)	10 (27.0%)	1 (2.7%)	13 (35.1%)	37	3.58	0.31
Men	31 (44.9%)	13 (18.8%)	7 (10.1%)	18 (26.1%)	69	–	–
*Targeted radiation region*							
Brain	14 (26.4%)	14 (26.4%)	4 (7.5%)	21 (39.5%)	53	**14.05**^**b**^	**0.02**
ENT and eye	22 (52.4%)	7 (16.7%)	3 (7.1%)	10 (23.8%)	42	–	–
Other body region	8 (72.7%)	2 (18.2%)	1 (9.1%)	0	11	–	–
*Technology*							
Proton	29 (34.9%)	20 (24.1%)	5 (6.0%)	29 (34.9%)	83	**10.57**^**c**^	**0.01**
Photon	15 (65.2%)	3 (13.0%)	3 (13.0%)	2 (8.7%)	23	–	–
*RT duration [days]*	43.55 ± 5.64	43.48 ± 4.98	40.63 ± 8.58	43.35 ± 5.48	43.25 ± 5.48	0.66	0.58
**Single dose [Gy]**	2.00 ± 0.10	1.90 ± 0.15	2.00 ± 0.01	1.90 ± 0.10	1.95 ± 0.10	5.17	0.13
**Radiation dose [Gy]**	63.6 ± 8.02	62.52 ± 6.72	59.25 ± 12.74	62.26 ± 7.11	62.66 ± 7.90	0.745	0.53
*Olfactory identification*	11.47 ± 2.64	11.74 ± 2.63	11.43 ± 1.81	11.83 ± 1.79	11.63 ± 2.35	0.16	0.92
*Nasal surgery*							
Yes	7 (25.9%)	5 (18.5%)	3 (11.1%)	12 (44.4%)	27	5.81	0.11
No	37 (46.8%)	18 (22.8%)	5 (6.3%)	19 (24.1%)	79	–	–
*Nasal polyps*							
Yes	2 (40.0%)	0	0	3 (60.0%)	5	–	–
No	42 (41.6%)	23 (22.8%)	8 (7.9%)	28 (27.7%)	101	–	–
*Allergy rhinitis*							
Yes	10 (45.4%)	4 (18.2%)	1 (4.5%)	7 (31.8%)	22	0.53	0.95
No	34 (40.5%)	19 (22.6%)	7 (8.3%)	24 (28.6%)	84	–	–
*Allergy rhinosinusitis*							
Yes	4 (36.4%)	2 (18.2%)	1 (9.1%)	4 (36.4%)	11	0.78	0.96
No	40 (42.1%)	21 (22.1%)	7 (7.4%)	27 (28.4%)	95	–	–
*Olfactory phantosmia early*							
Yes	1 (50.0%)	0	0	1 (50.0%)	2	–	–
No	43 (41.3%)	23 (22.1%)	8 (7.7%)	30 (28.8%)	104	–	–
*Regular nasal spray*							
Yes	3 (30.0%)	3 (30.0%)	2 (20.0%)	2 (20.0%)	10	3.39	0.32
No	41 (42.7%)	20 (20.8%)	6 (6.3%)	29 (30.2%)	96	–	–
*Taste problem*							
Yes	14 (66.7%)	5 (23.8%)	1 (4.8%)	1 (4.8%)	21	**10.28**^**d**^	**0.01**
No	30 (35.3%)	18 (21.2%)	7 (8.2%)	30 (35.3%)	85	–	–
*Ophthalmic surgery*							
Yes	4 (26.7%)	5 (33.3%)	2 (13.3%)	4 (26.7%)	15	3.21	0.33
No	41 (44.0%)	18 (19.8%)	6 (6.6%)	27 (29.7%)	91	–	–
*Myopia*							
Yes	23 (35.9%)	16 (25.0%)	4 (6.3%)	21 (32.8%)	64	3.09	0.38
No	21 (50.0%)	7 (16.7%)	4 (9.5%)	10 (23.8%)	42	–	–
*Hyperopia*							
Yes	31 (49.2%)	14 (22.2%)	4 (6.3%)	14 (22.2%)	63	5.19	0.15
No	13 (30.2%)	9 (20.9%)	4 (9.3%)	17 (39.5%)	43	–	–
*Cataract*							
Yes	2 (40.0%)	2 (40.0%)	0	1 (20.0%)	5	–	–
No	42 (41.6%)	21 (20.8%)	8 (7.9%)	30 (29.7%)	101	–	–
*Glaucoma*							
Yes	4 (57.1%)	1 (14.3%)	1 (14.3%)	1 (14.3%)	7	–	–
No	40 (40.4%)	22 (22.2%)	7 (7.1%)	30 (30.3%)	99	–	–
*Chemical/dust exposure*							
Yes	20 (47.6%)	8 (19.0%)	4 (9.5%)	10 (23.8%)	42	1.97	0.61
No	24 (37.5%%)	15 (23.4%)	4 (6.3%)	21 (32.8%)	64	–	–
*Alcohol*							
Never	5 (33.3%)	5 (33.3%)	1 (6.7%)	4 (26.7%)	15	7.43	0.26
Occasionally	19 (38.0%)	8 (16.0%)	3 (6.0%)	20 (40.0%)	50	–	–
Several times per week	20 (48.8%)	10 (24.4%)	4 (9.8%)	7 (17.1%)	41	–	–
*Tobacco*							
Yes	31 (44.3%)	17 (24.3%)	3 (4.3%)	19 (27.1%)	70	4.06	0.26
No	13 (36.1%)	6 (16.7%)	5 (13.9%)	12 (33.3%)	36	–	–
*Tumor duration [months]*	24.70 ± 17.07	19.52 ± 3.65	19.38 ± 3.50	23.68 ± 7.48	22.88 ± 11.99	1.22	0.31
*Tumor surgery*							
Yes	26 (34.7%)	17 (22.7%)	7 (9.3%)	25 (33.3%)	75	5.09	0.16
No	18 (58.1%)	6 (19.4%)	1 (3.2%)	6 (19.4%)	31	–	–
*Chemotherapy history*							
Yes	3 (37.5%)	1 (12.5%)	0	4 (50.0%)	8	–	–
No	40 (41.7%)	21 (21.9%)	8 (8.3%)	27 (28.1%)	96	–	–
*Chemotherapy with current RT*							
Yes	20 (38.5%)	14 (26.9%)	4 (7.7%)	14 (26.9%)	52	1.51	0.66
No	24 (45.3%)	8 (15.1%)	4 (7.5%)	17 (32.1%)	53	–	–
*Metastases*							
Yes	14 (66.7%)	3 (14.3%)	0	4 (19.0%)	21	6.34	0.08
No	30 (35.7%)	19 (22.6%)	8 (9.5%)	27 (32.1%)	84	–	–
*Head trauma*							
Yes	2 (100%)	0	0	0	2	–	–
No	42 (40.4%)	23 (22.1%)	8 (7.7%)	31 (29.8%)	104	–	–
*Hypertension*							
Yes	23 (46.0%)	9 (18.0%)	4 (8.0%)	14 (28.0%)	50	1.2	0.79
No	21 (37.5%)	14 (25.0%)	4 (7.1%)	17 (30.4%)	56	–	–
*Diabetes*							
Yes	4 (57.1%)	2 (28.6%)	0	1 (14.3%)	7	–	–
No	40 (40.4%)	21 (21.2%)	8 (8.1%)	30 (30.3%)	99	–	–
*Asthma*							
Yes	5 (41.7%)	2 (16.7%)	0	5 (41.7%)	12	1.32	0.80
No	39 (41.5%)	21 (22.3%)	8 (8.5%)	26 (27.7%)	94	–	–
*Obesity*							
Yes	3 (30.0%)	3 (30.0%)	2 (20.0%)	2 (20.0%)	10	3.39	0.32
No	41 (42.7%)	20 (20.8%)	6 (6.3%)	29 (30.2%)	96	–	–
Food allergy							
Yes	4 (40.0%)	2 (20.0%)	2 (20.0%)	2 (20.0%)	10	2.63	0.46
No	40 (41.7%)	21 (21.9%)	6 (6.3%)	29 (30.2%)	96	–	–
*Medication* allergy							
Yes	6 (37.5%)	1 (6.3%)	2 (12.5%)	7 (43.8%)	16	4.34	0.20
No	38 (42.2%)	22 (24.4%)	6 (6.7%)	24 (26.7%)	90	–	–
*Depression*							
Yes	5 (31.3%)	4 (25.0%)	3 (18.8%)	4 (25.0%)	16	3.65	0.26
No	39 (43.3%)	19 (21.1%)	5 (5.6%)	27 (30.0%)	90	–	–
*Epilepsy*							
Yes	7 (41.2%)	4 (23.5%)	1 (5.9%)	5 (29.4%)	17	0.22	0.99
No	37 (41.6%)	19 (21.3%)	7 (7.9%)	26 (29.2%)	89	–	–
Antihypertension medications							
Yes	22 (50.0%)	8 (18.2%)	4 (9.1%)	10 (22.7%)	44	3.08	0.4
No	22 (35.5%)	15 (24.2%)	4 (6.5%)	21 (33.9%)	62	–	–
*Anticoagulation*							
Yes	9 (56.3%)	3 (18.8%)	2 (12.5%)	2 (12.5%)	16	3.72	0.25
No	35 (38.9%)	20 (22.2%)	6 (6.7%)	29 (32.2%)	90	–	–
*Analgesics*							
Yes	7 (53.8%)	1 (7.7%)	1 (7.7%)	4 (30.8%)	13	–	–
No	37 (40.2%)	22 (23.9%)	7 (7.6%)	26 (28.3%)	92	1.96	0.59
*PPI*							
Yes	8 (50.0%)	2 (12.5%)	1 (6.3%)	5 (31.3%)	16	1.17	0.84
No	35 (39.8%)	21 (23.9%)	7 (8.0%)	25 (28.4%)	88	–	–
Antidepressants/antipsychotics/anticonvulsants							
Yes	9 (33.3%)	7 (25.9%)	3 (11.1%)	8 (29.6%)	27	1.63	0.66
No	34 (43.6%)	16 (20.5%)	5 (6.4%)	23 (29.5%)	78	–	–

*Note.* If group sample size was less than 10, counts and percent of the variable would be described but statistical analyses would not be conducted in order to avoid misleading outcomes due to small sample sizes

^a^regarding age, both sensations group were younger than no sensations group and phosphenes group

^b^regarding radiation region, a higher percentage of patients in both sensation group received brain radiation than other body region radiation, whereas a higher percentage of patients in no sensation group received other body region radiation than brain region radiation

^c^regarding radiation technology, a higher percentage of patients in both sensation group received proton radiation than photon radiation, whereas a higher percentage of patients in no sensation group received photon radiation than proton radiation

^d^regarding taste problem, a higher percentage of patients in both sensation group had no taste problem than had taste problem, whereas a higher percentage of patients in no sensation group had taste problem than had no taste problem

According to the logistic regression models in Table 4, there were several predictors of “phantosmias”, “phosphenes” or both when “no sensation” was set as reference. With increasing age, there was a decreasing risk of experiencing phantosmias (OR = 0.94, *p* < 0.01) during the RT. Compared to participants without taste problems, those reporting a taste problem had a lower risk to experience phantosmias (OR = 0.17, *p* = 0.03), and to co-experience both sensations (OR = 0.08, *p* = 0.03). Compared to patients treated with photon RT, patients treated with proton RT had an approximately 7‑fold higher risk of experiencing phosphenes (OR = 6.54, *p* < 0.01), and an almost 9‑fold greater risk of co-experiencing both sensations (OR = 8.67, *p* = 0.02). In addition, patients who received RT in brain region (OR = 10.36, *p* < 0.01) or ENT-related region (OR = 7.58, *p* = 0.03) had a significantly higher risk of experiencing phosphenes compared to those who received RT in other body region.

**Table 4:** Logistic regression models of phosphenes and/or phantosmias during radiation therapy

	B	SE	Wald	*p*	Exp(B)	95%CI	
						LB	UB
*Model 1: Phantosmia vs. no phantosmia*							
Taste problem = Yes	−1.73	0.81	*4.52*	*0.03*	0.17	0.04	0.87
Taste problem = No	Reference						
Age	−0.07	0.02	11.92	< 0.01	0.94	0.90	0.97
*Model 2: Phosphene vs. no phosphene*							
Technology = Proton	1.88	0.60	9.90	< 0.01	6.54	2.03	21.06
Technology = Photon	Reference						
RT region = Brain	2.34	0.85	7.55	< 0.01	10.36	1.95	54.92
RT region = ENT-related region	2.03	0.90	5.05	0.03	7.58	1.30	44.31
RT region = Other body region	Reference						
*Model 3: Both sensations vs. no sensation*							
Technology = Proton	2.16	0.93	5.35	0.02	8.67	1.39	54.10
Technology = Photon	Reference						
Taste problem = Yes	−2.48	1.15	4.69	0.03	0.08	0.01	0.79
Taste problem = No	Reference						

*B* beta coefficient, *SE* standard error, *Wald* Wald chi-square statistic, *p* level of significance, *Exp(B)* Exponentiated beta coefficient, *95% CI* 95% confidence interval, *LB* lower bound, *UB* upper bound.

Stepwise method was used to select the most important predictors of phosphene or/and phantosmia during the radiation therapy

#### Factors that influence the intensity and hedonic ratings of phantosmias and phosphenes

A series of linear regression analyses were conducted to identify predictors for the intensity and hedonic ratings of phantosmias and phosphenes. History of dust/chemical exposure predicted lower intensity (B = −1.52, t = 2.50, *p* = 0.02) and higher pleasantness (B = 0.49, t = 2.30, *p* = 0.03) of the phantosmias during RT. Longer duration of disease (B = 0.11, t = 3.15, *p* < 0.01), food allergy (B = 2.77, t = 3.21, *p* < 0.01), and epilepsy (B = −1.50, t = 2.39, *p* = 0.02) positively predicted phosphene intensity. In addition, analgesics (B = 0.47, t = 2.84, *p* < 0.01) negatively predicted pleasantness of the phosphenes during RT (Table 5).

**Table 5:** Linear regression models of phantosmias/phosphenes intensity and hedonic ratings

	B	SE	t	*p*	95% CI	
					LB	UB
*Model 1: Phantosmia intensity*						
History of dust/chemical exposure = Yes	−1.52	0.61	−2.50	0.02	−2.75	−0.29
*Model 2: Phantosmia hedonic*						
History of dust/chemical exposure = Yes	0.49	0.21	2.30	0.03	0.06	0.93
*Model 3: Phosphene intensity*						
Tumor duration [months]	0.11	0.04	3.15	< 0.01	0.04	0.18
Food allergy = Yes	2.77	0.86	3.21	< 0.01	1.04	4.50
Epilepsy = Yes	−1.50	0.63	−2.39	0.02	−2.76	−0.24
*Model 4: Phosphene hedonic*						
Analgesics = Yes	0.47	0.17	−2.84	< 0.01	0.14	0.81

*B* beta coefficient, *SE* standard error, *t* statistical test used to determine the significance of the B coefficient, *p* level of significance, *95% CI* 95% confidence interval, *LB* lower bound, *UB* upper bound

Stepwise method was used to select the most important predictors of phosphene or/and phantosmia during the radiation therapy

## Discussion

We performed a prospective study to assess olfactory and visual sensations during treatment sessions in patients having proton and photon therapy of different target regions. All patients had a baseline assessment of their olfactory function. Generally, over half of the patients experienced at least one type of abnormal sensations during radiation therapy, with 51% of the patients experiencing phosphenes and 35% of the patients experiencing phantosmias. This indicates that phosphenes and phantosmias are common during radiation therapy, and phosphenes are more frequent than phantosmias. In addition, we also found that patients more often co-experienced both sensations (28%), in comparison to the number of patients with solitary experiences of phantosmias (7%) or phosphenes (23%). This implies a shared mechanism of the generation of phantosmias and phosphenes. However, we acknowledge the need for caution when interpreting the high frequency of reported phantosmias and phosphenes. There is a potential risk of information bias due to typical side effects of pharmacotherapies when patients read medication leaflets, which may have led to overreporting of these sensations.

### Characteristics of abnormal sensations during radiation therapy

Regarding the characteristics of phantosmias, the sensations reached a medium intensity and were mildly unpleasant. Almost 90% the patients were free from phantosmias after one RT session was complete. The medium intensity, low level of unpleasantness and the temporary perception partly explained why such abnormal sensations generally do not interrupt the treatment [6]. This information could be a reasonable explanation for the patients if they perceive abnormal smells during the radiation, which may be helpful to prevent them from being anxious or even interrupt the course of RT. The most typical odors were described as “chemical”, “metallic” or “burnt”. Ozone, plastic and other smells were sometimes reported. Similar smells have been observed in previous studies [11].

At least two potential mechanisms may explain these smells. First, the generation of ozone in the proximity of the radiation beams may be a source for some of the patients’ odor percepts. Ozone is known to have a “chemical” or “burnt” smell [12], which was consistent with the descriptions of some patients in our study. In addition, a recent publication also suggested that ozone concentrations did reach potentially detectable levels in the treatment room [13]. However, they also pointed out that (a) only traces of ozone were found in the polyvinyl chloride tube that was used to mimic the nasal passages during radiation, and (b) the concentrations there were too low to detect by human [13]. Thus, in addition to ozone alone, unspecific activation of the olfactory system by radiation beam appears to be the main factor in the generation of odor phantoms [5, 6]. Several studies on intracerebral stimulations support this possibility [14]. For example, electrical stimulations of the mid-dorsal insula evokes olfactory sensations in epileptic patients, including metallic and chlorine smells [15]. Patients with temporal lobe epilepsy also report the perception of burnt smells when an intracerebral electrical stimulation is applied to the olfactory sulcus [16].

Phosphenes generally reached a medium intensity and but were not unpleasant. In approximately 80% of the cases phosphenes disappeared completely after one radiation therapy finished. Similar to phantosmias, phosphenes seem not to disturb patients too much. Phosphenes were typically perceived as a flashily blue, white or purple light in both eyes, and yellow, silver, and pink colors were reported occasionally. These colors were in line with previous studies. The blue and purple colors may be due to the generation of Cherenkov light inside the eye during radiation therapy [4, 11, 17]. Cherenkov light is caused by visible photons that are produced when a charged particle travels through a transparent medium that is faster than the speed of light in that medium, and it usually appears as blue to purple. The Cherenkov light has also been captured by Tendler et al., in patients’ eyes during stereotactic radiotherapy in a recent study [4]. Other than blue and purple, patients also commonly perceive white light and sometimes reported other colors, which means that the Cherenkov light was not the sole source of this effect. Similar to the generation of odor phantoms, unspecific stimulation of the visual system may also play a role. For example, electrical stimulation of the striatum and the geniculo-calcarine tract evoked phosphene in 7 out of the 17 cases, with the color ranging from white to colored light [18]. As for the differences in color, the cause is unknown. However, the color variation may result from different orientations of the beam relative to the brain, which has been linked to varied color of phosphenes [11].

### Factors that predict the occurrences of phantosmias and phosphenes

Based on the Chi-square and logistic regression analyses, phantosmias and phosphenes are more common in patients with younger age, radiation region of the brain, proton technology and less frequent in patients with a taste problem. Consisted with previous study, we observed that patients with younger age had a higher risk of experiencing phantosmias and co-experiencing phantosmias and phosphenes during radiation therapy. This may be explained by the decreasing function of the sense of smell with aging [12, 17]. However, we did not find significant differences of olfactory function in patients with and without odor phantoms. Notably, we only used the Sniffin’ Stick olfactory identification test to measure olfactory function, which may not provide an exhaustive picture of the patients’ olfactory abilities [19]. Hence, for future studies it would be advisable to employ the measurement of odor thresholds.

Regarding targeted radiation regions, among patients who reported any of the abnormal sensations, or a co-occurrence of both sensations, a higher percentage of them were received radiation of the brain or ENT region radiation than radiation of other body regions. This implied that it is not radiation alone which produces phantosmias or phosphenes but that the brain needs to be activated to produce these percepts. This result is in line with previous studies [8]. Interestingly, we found that phantosmias and phosphenes occurred more often in patients with brain irradiations than ENT region irradiation, which implied that the generation of phantosmias and phosphene may involve more central neural mechanisms than peripheral mechanism. Last but not least, phantosmias and phosphene could be elicited with activation of areas that are not regarded to be part of the olfactory or visual network. For example, phantosmias occurred in 2 out of 7 cases who were irradiated in the occipital lobe, a structure outside of typical olfactory eloquent areas. Phosphenes occurred in 8 out of 10 cases whose target region was in temporal lobe, a structure outside of typical visual areas. In addition, although there may be a risk of overreporting, phantosmias and phosphenes occurred in 1 and 2 out of 11 cases who had radiotherapy of prostate cancer, far away from olfactory/visual organs and neural systems. Although the mechanism was uncommon and unclear, this observation coincided with the perspective that “no neuron is an island”. The different brain regions are intensively connected, communicate and interact [20].

With regard to the type of RT used we found that patients who received proton therapy were more likely to report phantosmias and phosphenes than those received photon irradiation. Although the reason is unknown, it could be due to the interaction between the proton radiation and the irradiated region. Our results suggested that the brain region was strongly associated with a higher risk of experiencing abnormal sensations during RT, and it is notable that more patients receiving brain radiation therapy were treated with proton technology. This coincidence may partly explain why patients treated with proton RT reported higher abnormal sensations. However, it is possible that the difference in beam direction, energy delivery type of proton and photon technology could also contribute to the observed differences, which requires further investigation in future studies.

Interestingly, taste problems seemed to decrease the risk of having phantosmias and phosphenes during the radiation therapy. Although the underlying mechanism is unclear, taste problems (dysgeusia) are a common side effect of oncologic management, and it associates with a high risk of other neurosensory deficits, such as olfaction [21, 22]. Those who had a taste problem may also have a generally decreased chemosensory sensation, which might limit the patients’ responsiveness to olfactory or visual stimulation. However, we could not exclude the possibility that the “protective effect” of the taste problem could be due to the interactive effect with the location of the irradiated tumor. In the present set of data, patients with taste problems largely received a radiotherapy of the ENT region, while most patients without taste problems received irradiation in brain regions. Because irradiation of brain region is more strongly related to a higher risk of experiencing abnormal sensations during radiation therapy, this coincidence might explain the lower level of phantoms in patients with taste problems.

### Factors that predict the intensity and hedonic of phantosmias

Regression analyses indicated that patients with history of chemical/dust exposure are more likely to experience less intensive and less unpleasant phantosmia. It seemed that chemical/dust exposure decrease patients’ sensitivity and increase the tolerance towards phantosmia. Most of these patients were exposed to potentially toxic chemicals or dusts (e.g., exhaust gas, dyes) due to their work environment. On one hand, exposure to toxic chemical/dust could damage olfactory function [23–25]. On the other hand, long-term or frequent stimulation of the olfactory system may decrease the effectiveness of olfactory processes, which is so-called adaptation [26]. This would then be associated with a decrease of perceptual intensity [27, 28] and a change in pleasantness [29].

### Factors that predict the intensity and hedonic of phosphenes

Food allergies and longer duration of disease predicted a higher intensity of phosphenes. Patients with allergies may be more attentive towards their environment and their experiences for potentially harmful events. In fact, patients with allergies have been shown to have a high responsiveness in terms of bronchial symptoms, for example to harmless odorous stimuli [30]. This might have contributed to the perception of a higher intensity of the sensations.

In contrast, epilepsy was found to predict a lower perceived intensity of phosphenes. Epilepsy arises due to an imbalance between excitation and inhibition, which can cause hyperexcitability of neurons and hypersynchrony of neural circuits [31]. The additional stimulation from radiation may cause a desensitization effect, where neural circuits, including the visual pathway, become less responsive to further stimulation such as radiation, resulting in a lower intensity of phosphenes. However, further research is necessary to fully investigate the relationship between epilepsy and phosphenes during radiation therapy. Regression analysis also found that analgesics predicted lower unpleasantness of phosphenes. However, the reason remains unclear.

To some extent, the relationships between taste problem, history of chemical/dust exposure, food allergy, epilepsy, and phantosmias/phosphenes during radiation therapy seem to reveal that the overall sensitivity or arousal levels of the organism is related to the perception of such abnormal phenomena.

Several limitations and future plans should be pointed out. We examined the relationship between the targeted RT region, the average irradiation dose in the targeted RT region, and abnormal sensations. However, we did not contour olfactory eloquent areas or typical visual areas in the treatment planning or analyze them in the dose-volume histograms. It is important to note that the radiation technique and treatment planning can impact the dose deposition in adjacent areas, which could potentially activate phosphenes or phantosmias. For example, radiation that targeted the frontal lobe may also influence the olfactory bulb, probably with a reduced dose. Therefore, future studies should include precise dose-volume calculations for different regions to better understand the relationship between radiation therapy and abnormal sensations [32]. Apart from that our results implied an association between sensitivity or the level of arousal and the experience of phantosmias or phosphenes during radiation therapy, but more direct evidence is needed in future studies.

## Conclusion

Phantosmias and phosphenes are frequent. They often occur together during radiation therapy. Such abnormal sensations are more common in younger age, during irradiation region of the brain, and with proton therapy. The treatment settings and individual arousal level influence the occurrence, intensity and pleasantness of such abnormal sensations. Phantosmias and phosphenes may involve more central neural mechanism than peripheral mechanism. They can be elicited with activation of areas that are not regarded to be part of the olfactory or visual network.
